# Cancer Risk Near a Polluted River in Finland

**DOI:** 10.1289/ehp.6741

**Published:** 2004-04-15

**Authors:** Pia K. Verkasalo, Esa Kokki, Eero Pukkala, Terttu Vartiainen, Hannu Kiviranta, Antti Penttinen, Juha Pekkanen

**Affiliations:** ^1^Department of Environmental Health, National Public Health Institute, Kuopio, Finland; ^2^Finnish Cancer Registry, Institute for Statistical and Epidemiological Cancer Research, Helsinki, Finland; ^3^Department of Mathematics and Statistics, University of Jyväskylä, Jyväskylä, Finland

**Keywords:** cancer, dioxins, epidemiology, GIS, PCDD, PCDF, record linkage

## Abstract

The River Kymijoki in southern Finland is heavily polluted with polychlorinated dibenzo-*p*-dioxins and dibenzofurans and may pose a health threat to local residents, especially farmers. In this study we investigated cancer risk in people living near the river (< 20.0 km) in 1980. We used a geographic information system, which stores registry data, in 500 m × 500 m grid squares, from the Population Register Centre, Statistics Finland, and Finnish Cancer Registry. From 1981 to 2000, cancer incidence in all people (*N* = 188,884) and in farmers (*n* = 11,132) residing in the study area was at the level expected based on national rates. Relative risks for total cancer and 27 cancer subtypes were calculated by distance of individuals to the river in 1980 (reference: 5.0–19.9 km, 1.0–4.9 km, < 1.0 km), adjusting for sex, age, time period, socioeconomic status, and distance of individuals to the sea. The respective relative risks for total cancer were 1.00, 1.09 [95% confidence interval (CI), 1.04–1.13], and 1.04 (95% CI, 0.99–1.09) among all residents, and 1.00, 0.99 (95% CI, 0.85–1.15), and 1.13 (95% CI, 0.97–1.32) among farmers. A statistically significant increase was observed for basal cell carcinoma of the skin (not included in total cancers) in all residents < 5.0 km. Several other common cancers, including cancers of the breast, uterine cervix, gallbladder, and nervous system, showed slightly elevated risk estimates at < 5.0 km from the river. Despite the limitations of exposure assessment, we cannot exclude the possibility that residence near the river may have contributed to a small increase in cancer risk, especially among farmers.

The River Kymijoki is one of the largest rivers in southern Finland, with nearly 190,000 people living < 20.0 km from its shoreline (Figure 1). The river flows south to the Gulf of Finland, which is a part of the Baltic Sea surrounded by nine European countries. The effluents from several pulp and paper mills as well as from manufacturing of chloro alkali chemicals—in particular, a chlorophenol fungicide, Ky-5, in one factory—heavily loaded the river between the 1950s and the 1990s. The discharge of these compounds decreased during the 1990s after improvements in methods of pulp bleaching and effluent treatment and the ceasing of production in 1984 of Ky-5. However, the river sediments still contain high levels of the persistent and toxic environmental pollutants polychlorinated dibenzo-*p*-dioxins (PCDDs) and poly-chlorinated dibenzofurans (PCDFs) (Finnish Environment Institute 2003; Paasivirta 1996; Verta et al. 1999). The surface sediment levels of PCDD/Fs are between 0.5 and 350 ng/g in dry weight as toxic equivalents and thus are among the highest sediment levels observed worldwide. Elevated PCDD/F concentrations have also been measured in sediments of the Gulf of Finland (Isosaari et al. 2002), in fish caught from the River Kymijoki and the Gulf of Finland (Korhonen et al. 2001), and in fishermen living in the delta area (Kiviranta et al. 1999, 2002; Korhonen et al. 2001).

The most toxic congener, 2,3,7,8-tetra-chlorodibenzo-*p*-dioxin (2,3,7,8-TCDD), has also been classified by the International Agency for Research on Cancer (IARC) as “carcinogenic to humans” on the basis of sufficient evidence from animal and limited evidence from human studies (IARC 1997). For the other PCDD/Fs, there is inadequate or limited evidence of carcinogenicity from animal studies, and practically no studies have been conducted in humans. Overall, the strongest epidemiologic evidence for the carcinogenicity of 2,3,7,8-TCDD is for all cancers combined rather than for any specific site. The literature suggests an increase of 40% at most, deriving primarily from studies of occupational cohorts with mixed exposures (Kogevinas 2000; Kogevinas et al. 1997) and the industrial accident in Seveso, Italy (Bertazzi et al. 2001; Warner et al. 2002).

In this study we investigated cancer risk in people living near the River Kymijoki (< 20.0 km) using small-area statistics on health (SMASH) system designed for investigations of cancer risk near geographically defined exposure sources in Finland (Kokki et al. 2001). We assumed that PCDD/Fs are mobilized from the river surface sediments and reach nearby residents via the food chain (e.g., by consumption of locally caught fish). We hypothesized that cancer risk increases with decreasing distance to the river. Furthermore, we hypothesized that farmers show a higher risk than most other people, because farmers are more likely to be exposed to river water because of their lifestyles and/or because comparisons within a defined population group are less likely to be confounded.

## Materials and Methods

### Small-Area Statistics on Health System

The SMASH system has previously been used to investigate cancer risk near geographically defined exposure sources in Finland (Kokki et al. 2001, 2002; Pekkanen et al. 1995). It is a geographic information system (GIS) developed through a collaboration of the Department of Environmental Health, National Public Health Institute, Finland, and the Finnish Cancer Registry. The system runs on ArcView GIS, version 3.2 (Environmental Systems Research Institute Inc., Redlands, CA, USA) and stores nationwide registry data, in 500 m × 500 m grid squares, from the Population Register Centre, Statistics Finland, and the Finnish Cancer Registry. Data include population counts by age, sex, socioeconomic status (SES), and location coordinate of residence for 1980 and all cancer cases from 1981 to 2000).

All three source registries contain nationwide data with good quality and coverage. The Finnish Cancer Registry, founded in 1952, receives information on all known cases of cancer from hospitals, pathological and hematologic laboratories, and practicing physicians. A validation study showed that over 99% of all malignant cancers are registered by the Finnish Cancer Registry (Teppo et al. 1994). In 1999, cancer diagnoses were based on histologic confirmation in 94.6% of cases and solely on death certificates in 0.9% of cases (Finnish Cancer Registry 2003). A total of 27 cancers were selected to be studied. They were classified traditionally according to the International Classification of Diseases, 7th revision, [World Health Organization (WHO) 1995] modified by the Finnish Cancer Registry and include the most common cancer types and others that are of special interest in the case of PCDD/Fs. Basal cell carcinomas (BCCs) of the skin were not included in the total numbers because there are large variations in the BCC rates by hospital catchment area, suggesting that many cases may remain undetected. Nervous system tumors denote tumors of the central as well as the peripheral nervous system. Extranodal non-Hodgkin lymphomas were classified according to their primary site. The original data sets were linked using personal identification numbers unique to every resident in Finland. The data were available in 500 m × 500 m grid squares and were further aggregated according to our hypothesis on geographic reference to the river.

### Exposure Assessment

The study population was defined as all people (farmers in particular) living within 20.0 km from the River Kymijoki (i.e., in a 500 m × 500 m grid square at least partially located within 20.0 km from the river shoreline) on 31 December 1980. The correct registration of the place of residence (97% of Finns surveyed actually lived in the same building as that recorded in the registry) (Statistics Finland 1994) and the accurate geocoding of the latitude and longitude of the central points of each residence (± 10 m) ensure the correct spatial registration of cases and reference population relative to exposure sources of interest.

**Figure 1:**
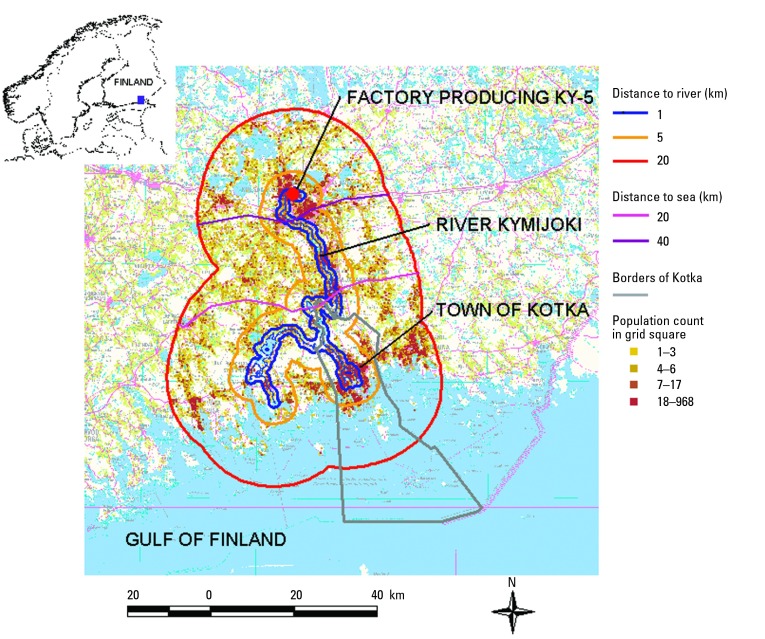
Exposure zones around the River Kymijoki. Reproduced with permission of the National Land Survey of Finland.

To allow comparisons within the study population, the study area was divided into nine subareas according to increasing distance to the river downstream from the factory producing Ky-5 (< 1.0 km; 1.0–4.9 km; 5.0–19.9 km), and according to increasing distance to the sea (< 20.0 km; 20.0–39.9 km; 40.0–59.9 km) (Figure 1). The cut points were selected *a priori* to distinguish varying exposure levels but remain, however, hypothetical. According to our primary hypothesis, the people and especially the farmers living nearest the river were suspected to be at the highest risk for cancer risk. The distance to sea variable was intended to measure pollution along the river flow on the north–south axis. However, its meaning is somewhat speculative. For example, many fish samples have been more heavily contaminated with PCDD/Fs close to the Gulf of Finland, but conversely, the surface sediments reach their peak levels near the factory producing Ky-5. SMASH was used to organize geographically defined data sets. The data sets were then entered into the SAS OnlineDoc statistical software, version 8 (SAS Institute Inc., Cary, NC, USA 1999) for estimation of cancer risk.

### Statistical Analyses

We assessed the risk for total cancer and 27 selected cancer types for all people, and separately for farmers, living near the river on 31 December 1980. All variables were classified according to the situation in 1980 and available in 500 m × 500 m grid squares. The total number of inhabited grid squares in 1980 was 197,520 for all of Finland and 4,687 for the study area.

For each grid square in Finland, numbers of subjects (population at risk in 1980) and observed cancers were counted by sex, age (5 years of age groups), time period (1981–1990–1991–2000), and SES (upper-level clerical workers, lower-level clerical workers, skilled workers, unskilled workers, farmers, unknown). For the study area, we counted numbers of subjects and observed cases of cancer according to distance between river and residence (< 1.0 km, 1.0–4.9 km, 5.0–19.9 km) and according to distance between sea and residence (< 20.0 km, 20.0–39.9 km, 40.0–59.9 km). We estimated reference incidence rates separately for total Finnish population and Finnish farmers, dividing the number of new cases of cancer by the population at risk in 1980, by sex, age, time period, and SES (in analyses of all people but not farmers). For the study area, we calculated expected numbers of cancers as the number of subjects multiplied by reference incidence rate for that cancer by sex, age, time period, SES, distance to sea, and distance to river.

Standardized incidence ratios (SIRs) were calculated by dividing the observed number of cases by the expected number of cases. SIRs were counted overall and by sex, age, SES (in analyses of all residents but not farmers), time period, distance to sea, and distance to river.

For distance to river comparisons, we used Poisson regression main-effect models for the observed numbers of cases in 3 × 3 contingency tables, where the classification is based on distance to river (three categories) and distance to sea (three categories). Logarithmically transformed expected numbers, formed from the reference population, were used as offset variables. We assumed that the sex, age, and SES, together with geographic effects related to river and sea, address the spatial variation in the data properly and give an interpretation in terms of distances. We plotted the residuals from the models for total cancer among all people and farmers and from models for BCCs among all people by distance to river and distance to sea. The model fits well with the data at the level of aggregation used. Detailed spatial analysis would be possible in theory (e.g., with Poisson regression with a correlated random component) (Best 1999) but would require partition of the study area into finer units and would likely be uninformative because of the small numbers.

We obtained maximum likelihood estimates for relative risk (RR) using PROC GENMOD in statistical software SAS (SAS Institute Inc.). The 95% confidence intervals (CIs) for SIRs are based on Poisson variation around expected values; confidence intervals for relative risk are two-sided Wald CIs. Statistical significance was set at *p* < 0.05.

## Results

In total, 188,884 people were living closer than 20.0 km from the River Kymijoki in 1980 (Table 1). Of these, 83% were < 60 years of age, 6% were farmers, 53% were living < 20.0 km from the sea, and 27% were living < 1.0 km from the river.

### Risk in All Residents

A total of 14,242 cases of cancer were diagnosed among the study cohort between 1981 and 2000. The incidence of total cancer in all residents was very similar to the general population risk (SIR = 0.99; 95% CI, 0.98 –1.01) (Table 2). Similarly, when studied by sex, age, or time period, the risk for total cancer differed no more than 3% from the general population risk. There was a subtle increase in the risk for total cancer in those living < 20.0 km and decreases in those living farther away from the Gulf of Finland. The SIRs for the 27 cancer subtypes studied were between 0.76 and 1.21 when comparing all residents with general population (Table 3). Statistically significant risk increases were observed for skin cancers.

The SES-adjusted relative risks for total cancer were 1.04 for those living < 1.0 km (95% CI, 0.99–1.09) and 1.09 for those living 1.0–4.9 km from the river (95% CI, 1.04–1.13), compared with those living 5.0–19.9 km from the river (Table 2). Overall, the SES-adjusted relative risks for total cancer suggested subtle increases between 1 and 15%, when analyzed by background variables. The relative risks were slightly higher for those living 1.0–4.9 km from the river than for those living < 1.0 km from the river. For those living 1.0–4.9 km from the river, relative risks for total cancer were statistically or marginally significantly increased in all subgroups but one (Table 2).

As for cancer subtypes, statistically significant risk increases were observed for BCC in those living < 1.0 km from the river and for cancers of the uterine cervix and corpus, breast, and lung and for BCCs in those living 1.0–4.9 from the river (Table 3). Several other cancer types also showed elevated risk estimates.

### Risk in Farmers

Between 1981 and 2000, a total of 1,143 cases of cancer were diagnosed among farmers living in the study area in 1980. The incidence of total cancer in farmers living in the study area did not differ statistically significantly from the incidence in all farmers in the country (RR = 0.96; 95% CI, 0.91–1.02) (Table 4). However, the risk was slightly decreased in men and in those living 40.0–59.9 km from the sea. A statistically significant risk increase was observed for liver cancer, and statistically or marginally significant risk decreases were observed for cancers of the stomach and lung.

The relative risk for total cancer in farmers was highest for those living < 1.0 km from the river (RR = 1.13; 95% CI, 0.97–1.32) (Table 4). The relative risks for total cancer in farmers living < 1.0 km from the river showed increases between 8 and 54% for all categories, although statistically significant increases were not detected. The highest estimate for relative risk (54% increase) was for those < 45 years of age at baseline. No consistent risk increases were observed for farmers living 1.0–4.9 km from the river.

No statistically significant risk changes by distance to river were observed for any of the 24 cancer subtypes for which the models converged (Table 5). However, for farmers living < 1.0 km from the river, the risk estimates for 14 subtypes were elevated by > 5%; the risk estimates for 4 were within 5% from reference; and those for 8 were decreased by > 5%. The respective numbers were 13, 2, and 9 for farmers living 1.0–4.9 km from the river.

## Discussion

### Small-Area Statistics on Health System

SMASH has been a useful tool in assessing cancer risks in freely selected areas in Finland (Kokki et al. 2001, 2002; Pekkanen et al. 1995). The high quality of nationwide registries on population and cancer (Teppo et al. 1994) also provided an excellent opportunity for the present study. The accurate geocoding of places of residence (± 10 m) contrasts SMASH with systems developed in many other countries (Aylin et al. 1999; National Cancer Institute 2003; National Center for Health Statistics 2003). Adjustment for SES was important, as socioeconomically determined lifestyle variations in risk can easily be attributed to environmental pollutants. In addition, the ability to use the most representative reference population (e.g., comparing farmers with farmers) further reduced the potential effects of confounding due to factors not related to the local environment. On the other hand, limitations of the methodology include the estimated denominators of the risk estimates (based on number of subjects in each 500 m × 500 m grid square in 1980), the small numbers for many cancer subtypes, and most importantly, the nonspecificity of exposure assessment.

### Exposure Assessment

In this study, exposure assessment was based solely on the place of residence at one point in time. In other words, we calculated the distance between residence and river shoreline but had no specific measure for PCDD/F exposure. To our knowledge, there are no previous GIS studies that have examined disease risks along a river. However, similar methodologies have been used to study risks close to other line-shaped features such as roads (Harrison et al. 1999), railways (Dickinson et al. 2003), and power lines (Feychting and Ahlbom 1993; Verkasalo et al. 1993). In PCDD/F epidemiology, GIS-based methodologies have previously been applied to detect cancer clusters around a municipal waste incinerator with high PCDD/F emissions (Viel et al. 2000) and to model airborne exposures to PCDD/Fs (Cohen et al. 2002; Floret et al. 2003; Stellman et al. 2003).

Possible health threats related to individuals living near this polluted river are an important issue for both decision makers and the general public. However, the use of a nonspecific surrogate measure for exposure may have introduced considerable measurement error or confounding by correlated exposures. To be considered a confounder, this other (correlating) exposure must be associated with individuals living near the river, and it would also have to show an association with increased risk of total cancer.

During the first half of the study period (as well as during several decades before that), the River Kymijoki was severely loaded with effluents from pulp bleaching and chloro alkali and Ky-5 manufacture, resulting in high environmental levels of polychlorinated phenols, catechols, guaiacols, PCDD/Fs, diphenyl ethers, and mercury (Paasivirta 1996). Of these pollutants, 2,3,7,8-TCDD has shown perhaps the strongest association with increased cancer risk (classified into group 1 by IARC) (IARC 2003). However, < 0.5% of total PCDD/F levels, measured as toxic equivalents, was explained by 2,3,7,8-TCDD (Vartiainen T, unpublished data). Other pollutants such as polychlorophenols (IARC group 2B: “possibly carcinogenic to humans”) and methyl mercury (IARC group 2B) may also be linked with increases in specific cancer subtypes. In practice this means that alternative or simultaneous effects of correlating environmental exposures cannot be excluded. Similarly, the possibility of a chance effect, residual confounding by some SES-related lifestyle factor, or confounding by some unidentified factor cannot be ruled out.

### Regional Variation in Cancer

Total cancer incidence in all people living < 20.0 km from the River Kymijoki was at the level expected based on the general population, whereas some particular cancer subtypes showed small increases or decreases in risk. In many cases observed cancer patterns may reflect commonly known reasons for regional variation in cancer.

For example, total cancer incidence in people living < 20.0 km from the Gulf of Finland was slightly increased compared with general population incidence but reflected the incidence in the town of Kotka (data not shown). In addition, the cancer pattern in the 20.0-km zone reflected increased SIRs for cancers of the bladder, pancreas, and skin (but no change for cancers of the stomach, lung, breast, and prostate) in Kotka (data not shown). This is no surprise, because 58% of the population living < 20.0 km from the sea lived precisely in the town of Kotka. Conversely, increased risk for bladder cancer, for example, has been associated with chlorination by-products (Koivusalo et al. 1997), which occur in high levels in the local municipal drinking water (Vartiainen T, unpublished data). Such exposures can prevent detection of an association between living close to the river and increased cancer risk (if such an association exists).

In this study the 23% increase for BCCs in people living < 20.0 km from the Gulf of Finland (data not shown) may reflect the generally high detection rates for BCC in the local hospital catchment area (30% higher incidence in men and 26% higher incidence in women compared with national average rates between 1995 and 1999; calculated based on the Finnish Cancer Registry data). These examples suggest that one should probably place more emphasis on local rather than on countrywide reference populations while using a GIS-based approach.

### Increased Risk?

In this study we found that cancer incidence in all people as well as in farmers living close to the River Kymijoki was at the level expected based on national rates. Among all people and farmers living < 1.00 km from the river, however, the SES-adjusted relative risks for total cancer were consistently > 1.00 (statistically nonsignificant), whether analyzed by sex, age, time period, or distance to sea. The lowest estimate for relative risk was 1.01 (for all residents ≥ 60 years of age at baseline); the highest estimate was 1.54 (for farmers < 45 years of age at baseline). The relative risks for farmers were generally higher than the relative risks for all residents. The relative risks for all people were also elevated <1.0–4.9 km from the river.

The magnitude of the effect was thus smaller than the effects described in earlier studies of the occupationally exposed PCDD/ PCDF cohorts (40% increase) (Kogevinas 2000; Kogevinas et al. 1997) and in the study of the Seveso cohort (30% increase) (Bertazzi et al. 2001). However, occupational cohorts tend to show higher risks than general population cohorts.

In principle, the suggestive increase in a broad spectrum of cancers is compatible with the consensus that the strongest epidemiologic evidence for the carcinogenicity of 2,3,7,8-TCDD is for all cancers combined, rather than for any specific site (IARC 1997). Traditionally, there are two clear examples of agents that cause an increase in cancers at many sites: tobacco (Baron and Rohan 1996) and ionizing radiation (Boice et al. 1996). Both, however, also show clearly elevated risks for some specific cancer subtypes. In the case of PCDD/Fs, it is not clear whether some specific cancer subtypes are more strongly associated with the exposure than other subtypes.

We observed a statistically significant risk increase for BCCs among all residents living < 1.0 km from the river. We also observed increases for cancers of the uterine cervix and corpus, breast, and lung, and BCCs among those living 1.0–4.9 from the river. No statistically significant risk changes occurred in cancer subtypes among farmers. However, several rather common cancers showed somewhat elevated risk estimates. The subtypes with suggestive risk increases among all residents and among farmers include the cancers of the uterine cervix and corpus, breast, and gallbladder. Among farmers living < 1 km from the river, on the other hand, suggestive risk increases of at least 50% were observed for cancers of the thyroid, uterine cervix, ovary, gallbladder, rectum, and breast, Hodgkin disease, and non-melanoma of the skin.

Cancers of the reproductive, endocrine, and hematopoietic systems and soft tissue sarcoma have traditionally been of interest to PCDD/F researchers. Our increased risk estimate for breast cancer in women was compatible with other studies suggesting an increase in breast cancer (Warner et al. 2002). Although this study included very few exposed cancers of the hematopoietic system, another GIS-based study has reported 2.3-fold risk increase in non-Hodgkin lymphoma due to PCDD/F emissions from a solid waste incinerator (Floret et al. 2003). Another GIS study examined the spatial distribution of sarcomas and non-Hodgkin lymphomas around a municipal solid waste incinerator with high emission levels of PCDD/Fs, identifying highly significant clusters around the incinerator (Viel et al. 2000). On the other hand, it is also worth noticing that we observed an increased risk estimate for lung cancer. Studies of the occupationally exposed PCDD/Fs cohorts (Kogevinas 2001) and the Seveso cohort (Bertazzi et al. 2001), but not the Swedish Baltic Sea fishermen cohort (Svensson et al. 1995), have reported risk increases for lung cancer.

## Conclusions

This study cannot exclude the possibility that residence near the River Kymijoki may have contributed to a subtle increase in the risk of total cancer, especially among farmers. The limitations of the available data and analytical methods must be recognized. It is also vital to appreciate that this is a small area (ecologic) study, where exposure assessment is based solely on place of residence, and the possible biologic pathway is not clear. Thus, this study can provide only first approximations of risks and tell only a little about causality.

## Figures and Tables

**Table 1:** Distribution of people living < 20.0 km from the River Kymijoki in 1980 according to sex, age, SES, distance to sea, and distance to river.

Variable	No. (%)
Sex	
Men	91,687 (48.5)
Women	97,197 (51.5)
Age (years)	
< 15	37,013 (19.6)
15–29	44,974 (23.8)
30–44	41,621 (22.1)
45–59	33,704 (17.8)
≥60	31,572 (16.7)
SES	
Upper-level clerical workers	22,463 (11.9)
Lower-level clerical workers	40,004 (21.2)
Skilled workers	20,275 (10.7)
Unskilled workers	56,012 (29.7)
Farmers	11,132 (5.9)
Unknown	38,998 (20.6)
Distance to the Gulf of Finland (km)	
< 20.0	100,276 (53.1)
20.0–39.9	34,007 (18.0)
40.0–59.9	54,601 (28.9)
Distance to the River Kymijoki (km)	
< 1.0	51,723 (27.4)
1.0–4.9	82,243 (43.5)
5.0–19.9	54,918 (29.1)
Total	188,884 (100.0)

**Table 2:** Risk for total cancer between 1981 and 2000 among all people living < 20.0 km from the River Kymijoki in 1980.

	Distance to the River Kymijoki (km)										
	< 1.0			1.0–4.9			5.0–19.9		All people < 20.0		
Variable	Obs	RR	95% CI	Obs	RR	95% CI	Obs	RR	Obs	SIR*^a^*	95% CI
All sites	3,866	1.04	0.99–1.09	6,338	1.09	1.04–1.13	4,038	1.00	14,242	0.99	0.98–1.01
Sex											
Men	1,819	1.04	0.97–1.11	2,979	1.10	1.03–1.16	1,970	1.00	6,768	0.97	0.95–0.99
Women	2,047	1.04	0.97–1.10	3,359	1.08	1.02–1.14	2,068	1.00	7,474	1.01	0.99–1.04
Age (years)											
< 45	793	1.04	0.94–1.15	1,351	1.10	1.00–1.20	811	1.00	2,955	0.98	0.95–1.02
45–59	1,347	1.07	0.99–1.16	2,277	1.11	1.04–1.19	1,359	1.00	4,983	0.99	0.97–1.02
≥60	1,726	1.01	0.95–1.08	2,710	1.07	1.00–1.13	1,868	1.00	6,304	0.97	0.97–1.02
Time period											
1981–1990	1,738	1.04	0.97–1.11	2,746	1.07	1.01–1.14	1,825	1.00	6,309	0.97	0.95–1.00
1991–2000	2,128	1.03	0.97–1.10	3,592	1.10	1.04–1.16	2,213	1.00	7,933	1.01	0.98–1.03
Distance to the Gulf of Finland (km)											
< 20.0	2,140	1.03	0.97–1.09	3,175	1.07	1.02–1.12	2,816	1.00	8,131	1.04	1.02–1.07
20.0–39.9	795	1.02	0.92–1.14	952	1.06	0.96–1.18	510	1.00	2,257	0.92	0.88–0.96
40.0–59.9	931	1.09	0.98–1.20	2,211	1.15	1.06–1.26	712	1.00	3,854	0.93	0.91–0.96

Obs, observed number of cases.

**a**All people in Finland were used as reference for SIRs.

**Table 3:** Risks for cancer subtypes between 1981 and 2000 among all people living < 20.0 km from the River Kymijoki in 1980.

	Distance to the River Kymijoki (km)										
	< 1.0			1.0–4.9			5.0–19.9		All people < 20.0		
Primary site	Obs	RR	95% CI	Obs	RR	95% CI	Obs	RR	Obs	SIR*^a^*	95% CI
Larynx, epiglottis	31	1.64	0.93–2.89	35	1.15	0.66–2.00	20	1.00	86	0.88	0.70–1.09
Cervix uteri	39	1.61	0.97–2.67	61	1.65	1.03–2.64	25	1.00	125	1.02	0.85–1.22
Gallbladder, bile ducts	60	1.45	0.98–2.15	83	1.34	0.93–1.94	44	1.00	187	0.90	0.77–1.03
Corpus uteri	128	1.20	0.93–1.54	220	1.31	1.05–1.65	116	1.00	464	1.06	0.96–1.16
Lip	34	1.14	0.72–1.81	44	0.99	0.64–1.53	38	1.00	116	1.06	0.88–1.27
Skin, BCC*^b^*	1,000	1.13	1.03–1.24	1,566	1.13	1.04–1.23	953	1.00	3,519	1.16	1.13–1.20
Ovary	104	1.13	0.85–1.49	139	0.94	0.73–1.23	98	1.00	341	1.02	0.91–1.13
Breast	554	1.12	0.99–1.26	915	1.15	1.03–1.28	512	1.00	1,981	0.97	0.93–1.01
Nervous system	133	1.12	0.87–1.43	232	1.22	0.98–1.53	124	1.00	489	1.00	0.91–1.09
Rectum, rectosigmoid	162	1.09	0.88–1.36	272	1.16	0.95–1.41	156	1.00	590	1.05	0.97–1.14
Stomach	220	1.08	0.90–1.31	352	1.12	0.95–1.33	224	1.00	796	0.97	0.90–1.03
Skin, nonmelanoma	165	1.08	0.87–1.34	241	1.05	0.87–1.28	176	1.00	582	1.21	1.11–1.31
Multiple myeloma	45	1.08	0.72–1.63	76	1.18	0.81–1.70	47	1.00	168	0.88	0.76–1.03
Bladder, ureter, urethra	143	1.05	0.83–1.32	241	1.14	0.93–1.40	153	1.00	537	0.97	0.89–1.06
Non-Hodgkin lymphoma	102	1.03	0.78–1.35	164	1.04	0.81–1.33	104	1.00	370	1.02	0.92–1.13
Prostate	399	1.02	0.89–1.17	587	0.98	0.87–1.11	444	1.00	1,430	0.97	0.92–1.02
Lung, trachea	396	1.00	0.87–1.14	709	1.14	1.01–1.28	433	1.00	1,538	0.90	0.86–0.95
Liver	51	1.00	0.68–1.49	84	1.05	0.74–1.50	50	1.00	185	1.01	0.87–1.17
Kidney	144	0.98	0.78–1.23	231	0.99	0.81–1.22	159	1.00	534	1.06	0.97–1.15
Thyroid gland	45	0.97	0.64–1.45	75	1.00	0.69–1.44	49	1.00	169	0.76	0.65–0.88
Soft tissues	25	0.97	0.56–1.67	49	1.23	0.77–1.98	28	1.00	102	1.07	0.87–1.30
Skin, melanoma	103	0.93	0.72–1.22	204	1.17	0.93–1.47	119	1.00	426	1.12	1.01–1.23
Pancreas	145	0.91	0.73–1.14	257	1.05	0.87–1.28	171	1.00	573	1.07	0.99–1.16
Testis	14	0.91	0.44–1.85	27	1.10	0.59–2.04	17	1.00	58	1.16	0.88–1.49
Colon	221	0.88	0.73–1.05	390	0.99	0.84–1.16	269	1.00	880	1.05	0.98–1.12
Leukemia	72	0.84	0.62–1.15	141	1.06	0.82–1.38	96	1.00	309	0.96	0.86–1.08
Hodgkin disease	14	0.65	0.33–1.26	38	1.11	0.66–1.97	24	1.00	76	0.87	0.68–1.08

Obs, observed number of cases.

**a**All people in Finland were used as reference for SIRs.

**b**BCCs of the skin are not included in the total numbers.

**Table 4:** Risk for total cancer between 1981 and 2000 among farmers living < 20.0 km from the River Kymijoki in 1980.

	Distance to the River Kymijoki (km)										
	< 1.0			1.0–4.9			5.0–19.9		All farmers < 20.0		
Variable	Obs	RR	95% CI	Obs	RR	95% CI	Obs	RR	Obs	SIR*^a^*	95% CI
All sites	209	1.13	0.97–1.32	230	0.99	0.85–1.15	704	1.00	1,143	0.96	0.91–1.02
Sex											
Men	112	1.10	0.89–1.36	123	0.95	0.77–1.16	404	1.00	639	0.92	0.85–1.00
Women	97	1.16	0.92–1.46	107	1.04	0.83–1.29	300	1.00	504	1.01	0.93–1.11
Age (years)											
< 45	27	1.54	0.98–2.42	24	1.09	0.69–1.74	70	1.00	121	0.97	0.80–1.15
45–59	68	1.08	0.82–1.42	78	0.99	0.77–1.28	246	1.00	392	0.97	0.88–1.07
≥60	114	1.08	0.87–1.33	128	0.97	0.79–1.18	388	1.00	630	0.95	0.88–1.03
Time period											
1981–1990	100	1.15	0.92–1.45	115	1.08	0.87–1.34	322	1.00	537	0.96	0.88–1.05
1991–2000	109	1.11	0.89–1.38	115	0.91	0.74–1.13	382	1.00	606	0.96	0.88–1.04
Distance to the Gulf of Finland (km)											
< 20.0	112	1.11	0.89–1.38	113	1.16	0.93–1.44	279	1.00	504	1.01	0.92–1.10
20.0–39.9	67	1.11	0.85–1.46	81	0.90	0.70–1.16	244	1.00	392	0.99	0.90–1.10
40.0–59.9	30	1.33	0.91–1.96	36	0.80	0.56–1.14	181	1.00	247	0.83	0.73–0.94

Obs, observed number of cases.

**a**All farmers in Finland were used as reference for SIRs.

**Table 5:** Risks for cancer subtypes in 1981–2000 among farmers living < 20.0 km from the River Kymijoki in 1980.

	Distance to the River Kymijoki (km)										
	< 1.0			1.0–4.9			5.0–19.9		All farmers < 20.0		
Primary site	Obs	RR	95% CI	Obs	RR	95% CI	Obs	RR	Obs	SIR*^a^*	95% CI
Thyroid gland	3	2.29	0.54–9.69	3	1.70	0.41–7.14	5	1.00	11	0.79	0.39–1.41
Hodgkin disease	2	2.20	0.36–13.42	2	2.02	0.34–12.13	3	1.00	7	1.06	0.42–2.18
Cervix uteri	1	2.04	0.18–22.72	2	2.68	0.38–19.08	2	1.00	5	0.75	0.24–1.76
Ovary	9	1.83	0.81–4.12	2	0.32	0.07–1.39	18	1.00	29	1.24	0.83–1.78
Skin, nonmelanoma	15	1.72	0.92–3.19	5	0.47	0.18–1.21	32	1.00	52	1.04	0.78–1.37
Gallbladder, bile ducts	3	1.72	0.43–6.97	3	1.46	0.36–5.86	6	1.00	12	0.72	0.37–1.26
Rectum, rectosigmoid	13	1.65	0.86–3.18	7	0.69	0.30–1.56	31	1.00	51	1.07	0.80–1.41
Breast	23	1.54	0.94–2.52	24	1.28	0.79–2.07	55	1.00	102	0.92	0.75–1.11
Nervous system	8	1.36	0.60–3.07	11	1.48	0.72–3.06	22	1.00	41	1.29	0.92–1.75
Corpus uteri	8	1.27	0.56–2.87	8	1.01	0.45–2.26	23	1.00	39	1.17	0.83–1.60
Skin, BCC*^b^*	56	1.26	0.93–1.72	43	0.80	0.57–1.12	160	1.00	259	1.07	0.94–1.21
Stomach	12	1.22	0.63–2.35	17	1.37	0.77–2.43	38	1.00	67	0.79	0.61–1.00
Lung, trachea	19	1.13	0.68–1.89	26	1.18	0.75–1.85	70	1.00	115	0.78	0.64–0.93
Pancreas	9	1.07	0.51–2.27	11	1.05	0.53–2.10	31	1.00	51	1.14	0.85–1.50
Liver	4	1.01	0.33–3.08	6	1.19	0.46–3.08	15	1.00	25	2.17	1.41–3.21
Prostate	27	0.99	0.65–1.52	32	0.92	0.62–1.37	107	1.00	166	0.99	0.84–1.15
Bladder, ureter, urethra	7	0.91	0.39–2.08	6	0.63	0.26–1.52	30	1.00	43	0.86	0.62–1.16
Kidney	7	0.87	0.38–1.98	13	1.31	0.68–2.52	30	1.00	50	1.24	0.92–1.63
Leukemia	5	0.87	0.33–2.34	9	1.33	0.61–2.91	21	1.00	35	1.19	0.83–1.66
Non-Hodgkin lymphoma	4	0.70	0.24–2.04	3	0.40	0.12–1.35	22	1.00	29	0.99	0.66–1.42
Colon	5	0.45	0.18–1.15	10	0.73	0.36–1.46	40	1.00	55	0.88	0.66–1.15
Skin, melanoma	2	0.39	0.09–1.96	5	0.78	0.29–2.10	19	1.00	26	0.88	0.58–1.29
Multiple myeloma	1	0.39	0.05–3.10	3	1.09	0.29–4.11	8	1.00	12	0.66	0.34–1.16
Soft tissues	3	NC	NC	0	NC	NC	7	NC	10	1.29	0.62–2.37
Testis	1	NC	NC	0	NC	NC	2	NC	3	1.09	0.22–3.18
Larynx, epiglottis	2	NC	NC	0	NC	NC	4	NC	6	0.80	0.29–1.73
Lip	3	NC	NC	4	NC	NC	4	NC	11	0.59	0.59–1.06

Abbreviations: —, models for these cancer subtypes did not converge; NC, models for these cancer subtypes did not converge; Obs, observed number of cases.

**a**All farmers in Finland were used as reference for SIRs.

**b**BCCs of the skin are not included in total numbers.
